# Optimizing Diffusion Imaging Protocols for Structural Connectomics in Mouse Models of Neurological Conditions

**DOI:** 10.3389/fphy.2020.00088

**Published:** 2020-04-21

**Authors:** Robert J. Anderson, Christopher M. Long, Evan D. Calabrese, Scott H. Robertson, G. Allan Johnson, Gary P. Cofer, Richard J. O’Brien, Alexandra Badea

**Affiliations:** 1 Department of Radiology, Duke University, Durham, CA, United States,; 2 Department of Radiology and Biomedical Imaging, University of California, San Francisco, CA, United States,; 3 Department of Neurology, School of Medicine, Duke University, Durham, CA, United States

**Keywords:** diffusion imaging, MRI, connectivity (graph theory), mouse model, brain, neurodegenerative diseases

## Abstract

Network approaches provide sensitive biomarkers for neurological conditions, such as Alzheimer’s disease (AD). Mouse models can help advance our understanding of underlying pathologies, by dissecting vulnerable circuits. While the mouse brain contains less white matter compared to the human brain, axonal diameters compare relatively well (e.g., ~0.6 μm in the mouse and ~0.65–1.05 μm in the human corpus callosum). This makes the mouse an attractive test bed for novel diffusion models and imaging protocols. Remaining questions on the accuracy and uncertainty of connectomes have prompted us to evaluate diffusion imaging protocols with various spatial and angular resolutions. We have derived structural connectomes by extracting gradient subsets from a high-spatial, high-angular resolution diffusion acquisition (120 directions, 43-μm-size voxels). We have simulated protocols with 12, 15, 20, 30, 45, 60, 80, 100, and 120 angles and at 43, 86, or 172-μm voxel sizes. The rotational stability of these schemes increased with angular resolution. The minimum condition number was achieved for 120 directions, followed by 60 and 45 directions. The percentage of voxels containing one dyad was exceeded by those with two dyads after 45 directions, and for the highest spatial resolution protocols. For the 86- or 172-μm resolutions, these ratios converged toward 55% for one and 39% for two dyads, respectively, with <7% from voxels with three dyads. Tractography errors, estimated through dyad dispersion, decreased most with angular resolution. Spatial resolution effects became noticeable at 172 μm. Smaller tracts, e.g., the fornix, were affected more than larger ones, e.g., the fimbria. We observed an inflection point for 45 directions, and an asymptotic behavior after 60 directions, corresponding to similar projection density maps. Spatially downsampling to 86 μm, while maintaining the angular resolution, achieved a subgraph similarity of 96% relative to the reference. Using 60 directions with 86- or 172-μm voxels resulted in 94% similarity. Node similarity metrics indicated that major white matter tracts were more robust to downsampling relative to cortical regions. Our study provides guidelines for new protocols in mouse models of neurological conditions, so as to achieve similar connectomes, while increasing efficiency.

## INTRODUCTION

A growing body of evidence suggests that altered brain connectivity is present in a variety of neurologic and psychiatric diseases. For example neurological conditions such as AD [1, 2], autism spectrum disorders [3], and psychiatric conditions such as schizophrenia [4] can be viewed as connectopathies. Multimodal network analyses can help identify early alterations associated with pathological processes both at the functional and structural levels. Structural connectomes based on diffusion MRI may produce sensitive biomarkers to monitor these conditions since they integrate the effects of multiple pathologies (atrophy, myelination, toxicity of the environment due to, e.g., oligomers, microstructural changes) [5].

While studies on diffusion imaging in the human brain have previously addressed the tradeoffs associated with spatial vs. angular resolution, the mouse brain provides insight into white matter connectivity at a vastly different scale and, thus, deserves special attention. We need to compare 2-mm linear dimension voxel sizes in humans (corresponding to 8-mm^3^ voxel volumes), vs. 0.2–0.043-mm linear dimension voxel sizes (corresponding to ~8 × 10^−2^- 8 × 10^−5^-mm^3^ voxel volumes) required to distinguish similar levels of anatomical detail in mouse brains [6–11]. This increased resolution enables the observation of brain architecture at the level of cellular layers, which, therefore, can help us gain insight into the mechanistic drivers behind the etiology and progression of disease, using animal models where axonal dimensions are relatively similar to humans [12, 13].

Yet, using sensitive methods for identifying pathways affected early on in animal models remains difficult. Difficulties in optimizing diffusion imaging protocols arise from the need for long acquisition times, to achieve sufficient signal to noise for smaller voxels. These are required for dissecting vulnerable pathways and networks, early on in the disease process, or subtle changes in time or following interventions. The mouse brain architecture, in contrast with the human brain, presents with thin, relatively sparse white matter tracts. The imaging protocols required for resolving small tract-based connections are, thus, demanding a high spatial resolution. In addition, increasing the angular sampling and the number of b values may reduce biases and produce more accurate models. This translates into long protocols, and subsequently costs in terms of time and money, which need to be balanced against the desired increases in spatial and angular resolution.

In this work, we have examined the balance between spatial and angular resolutions and inferred suggestions for recommended future protocols. In particular, we examined a set of nodes/brain regions that are relevant for neurodegenerative conditions such as AD. We used simulations based on downsampling a high-spatial, high-angular data set, and assessed the effect of relaxing these requirements/parameters on the accuracy of the reconstructed tracts and connectome, when compared against our reference protocol. We have focused our analyses on regions expected to be affected in AD, such as fimbria, fornix, and hippocampus, septum, hypothalamus, and also the lateral geniculate nucleus. We evaluated how different protocols led to increasingly more robust results and how fast errors change with spatial and angular sampling resolution. Our results can inform future population studies, in particular, for models of neurodegenerative disease.

## METHODS

Imaging was performed, as previously reported [14] on a 9.4-Tesla small-animal imaging system controlled by an Agilent VnmrJ 4 console. Diffusion imaging was accomplished using a 3D diffusion-weighted spin-echo pulse sequence with repetition time (TR) = 100 ms, echo time (TE) = 15 ms, and b value = 4,000 s/mm^2^. The acquisition matrix was 568 × 284 × 228 over a 24.4 × 12.2 × 9.8 mm field of view, resulting in isotropic 43 × 43 × 43 μm voxels (referred from now on as 43-μm linear voxel dimension). The diffusion protocol included 120 diffusion directions [15, 16] and 11 non-diffusion-weighted (b0) measurements (i.e., one b0 every 12 diffusion measurements).

Angular downsampling of the original diffusion data set was performed by obtaining the optimal diffusion directions for each angular subset [15, 16] and extracting the closest gradient vector from the 120 unique diffusion directions. The closest gradient orientation was chosen by maximizing the dot product between the optimal gradient vector and the possible vectors found in the original gradient table.

Spatial downsampling of the original diffusion data set was performed by cropping the data in k space, resulting in three levels of isotropic spatial resolution: 43-, 86-, 172-μm linear voxel dimension.

The condition numbers for the gradient subset matrices, defining angular sampling, were calculated in MATLAB (Natick, MA), based on the ratio between the largest and smallest singular values for each matrix. The stability of the condition numbers was evaluated for each of the gradient subsets following 50,000 simulated rotations of these matrices.

Diffusion data processing was done on a high-performance computing cluster with 96 physical cores and 1.5 TB of RAM. All 131 image volumes were registered to the first b0 image using advanced normalization tools (ANTs) affine transformation [17] to correct for eddy current distortions. Scalar image volumes were reconstructed using FSL’s DTIFIT [18]. Fiber data for probabilistic tractography were reconstructed using FSL’s BEDPOSTX [19] with a maximum of four fiber orientations per voxel. In-home written scripts and FSL were used to estimate the numbers of tracts with one, two, three, or four dyads and to estimate errors/uncertainty based on dyad dispersion.

Automated atlas-based segmentation was performed [20] using an atlas, which combines the Waxholm Space atlas [21] for subcortical labels, and the Ullmann atlas of the neocortex [22], with a total of 332 regions—symmetrized relative to the midsagittal plane. Connectomes were constructed using SAMBA [23], and DSI Studio [24] for a subset of brain regions. These include for simplicity, the connectivity matrices generated pertaining solely to left-sided seed regions connecting to left-sided targets. We next evaluated the similarity of connectomes based on global Spearman correlation coefficients among 12 representative acquisition schemes (four angular sampling schemes and three spatial resolution levels). Node wise similarity was evaluated as in Blondel et al. [25]. Specifically, the similarity matrices were obtained as the limit of the normalized even iterates of *S*_*k*_ + 1 = *BS*_*k*_*A*^*T*^ + *B*^*T*^*S*_*k*_*A*, where *A* and *B* are the two graph adjacency matrices, and *S*_0_ is a matrix whose entries are equal to 1.

## RESULTS

We have evaluated the effect of different angular diffusion sampling schemes and spatial resolutions on mouse brain connectivity, from the point of view of stability, with reference to a published data set, and with the aim to identify balanced acquisition schemes in terms of cost and accuracy.

**FIGURE 1 |:**
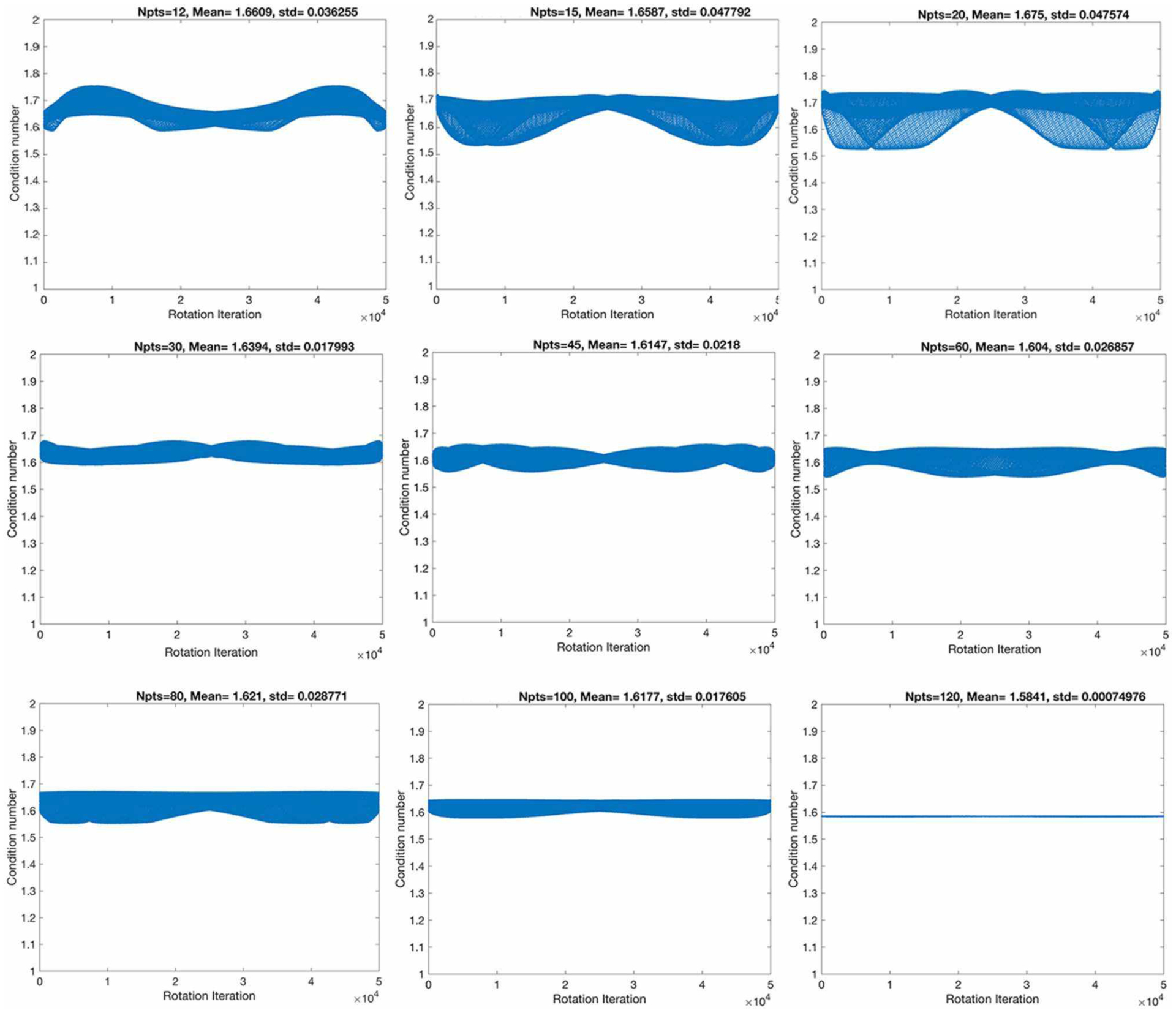
The stability of the condition numbers increased, and the standard deviation decreased with angular resolution, although not monotonically, denoting that some sampling schemes are more robust to rotational variance, due to their geometrical symmetry properties.

First, we have evaluated nine angular sampling schemes from the point of view of their stability, through their condition numbers, as well as their standard deviations (Figure 1). The condition numbers define the asymptotic worst-case relative change in output for a relative change in input.

The highest spatial and angular resolution scheme had the smallest condition numbers (1.58) and standard deviation (7.5 × 10^−4^). The ranking in terms of condition number was followed by the 60- and 45-direction angular sampling schemes, while the standard deviation followed the ranking 100, 45, and 60 (Table 1).

We focused the rest of our analyses on the 45- and 60- direction schemes and compared the results against the reference 120-direction scheme, at three different spatial resolution levels throughout.

**FIGURE 2 |:**
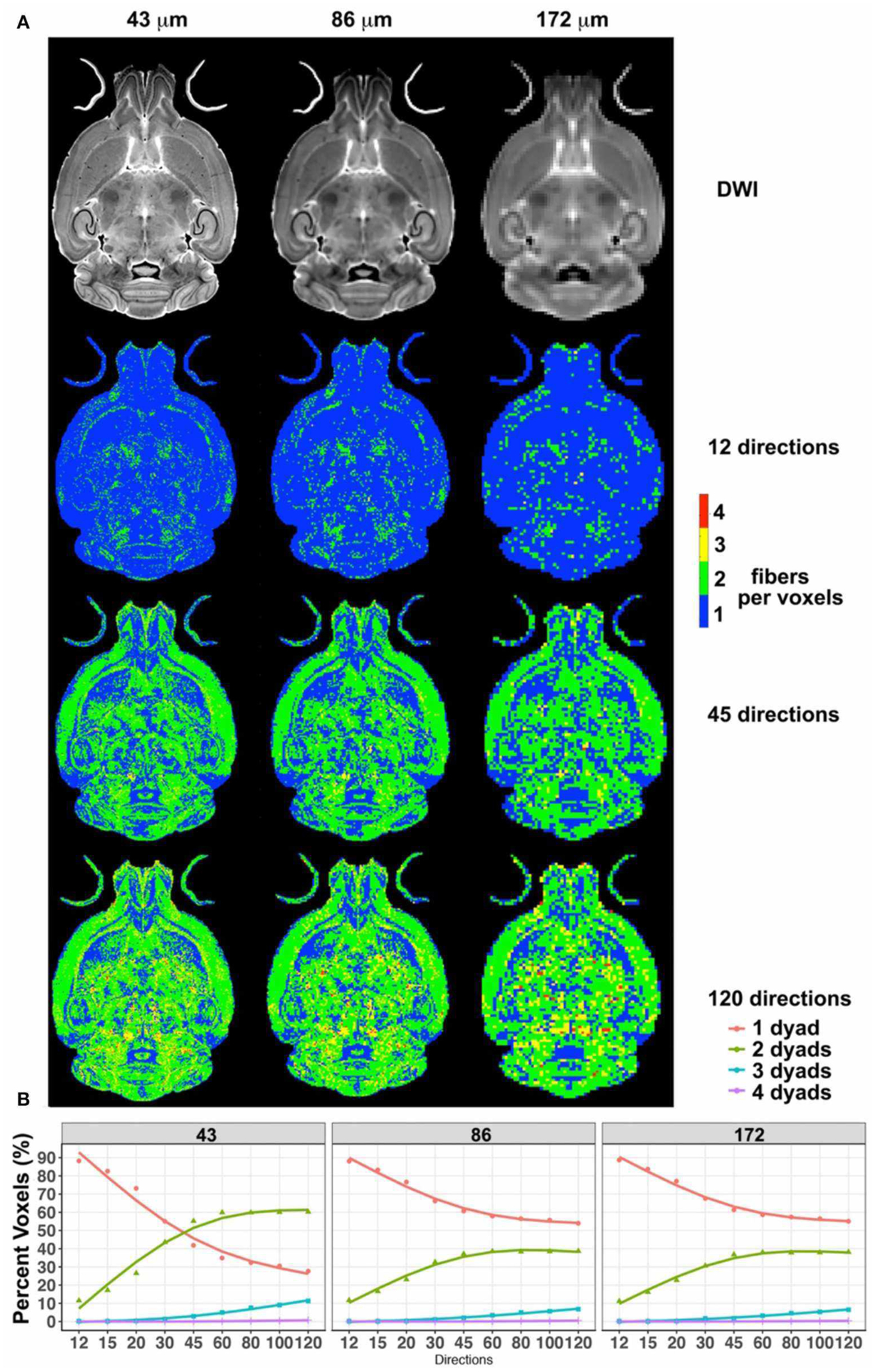
The effect of spatial resolution (horizontal axis) and angular resolution (vertical axis) on the number of voxels with one, two, three, or four fibers (dyads) per voxels **(A)**. **(A)** Effects for 12, 45, and 120 directions, chosen as examples for low-, medium-, and high-angular sampling. **(B)** Effects for sampling schemes between 12 and 120 at three spatial resolutions (43, 86, and 172 μm). These results illustrated the advantages of high-angular and spatial-resolution protocols in terms of sensitivity and stability.

Downsampled diffusion data sets were used to estimate the total number of fibers per voxel in each set (Figure 2). The total fiber count increased from 7.4 × 10^6^ for 12 directions to 1.07 × 10^7^ for 45 and 1.13 × 10^7^ for 60 directions and 1.23 × 10^7^ for 120 directions. The steepest changes occurred below 45 directions.

Interestingly, while the number of voxels detected to have one fiber direction was dominant for small angular sampling schemes, this number was exceeded by the number of voxels identified to have two fiber directions, if the sampling schemes had more than 45 angular directions and at a spatial resolution of 43 μm. The number of voxels with one dyad reached 42% for 45 directions, and 35% for 60 directions. The number of voxels with two dyads increased with increasing angular sampling, reaching 55% for 45 directions and 60% for 60 directions. Thus, these two curves intersected, and the ratio of one- to two-dyad voxels changed after ~45 directions. However, the two curves did not intersect for the 86- and 172-μm spatial resolution scenarios. The number of voxels with one fiber direction plateaued at ~55%, and the number of voxels with two fiber directions at ~38% for 86- and 172-μm spatial resolutions, with modest increases between 45 and 60 angular samples, and <7% contribution from voxels with three directions. These two data sets had very similar behaviors in terms of the number of voxels with one or two dyads.

**FIGURE 3 |:**
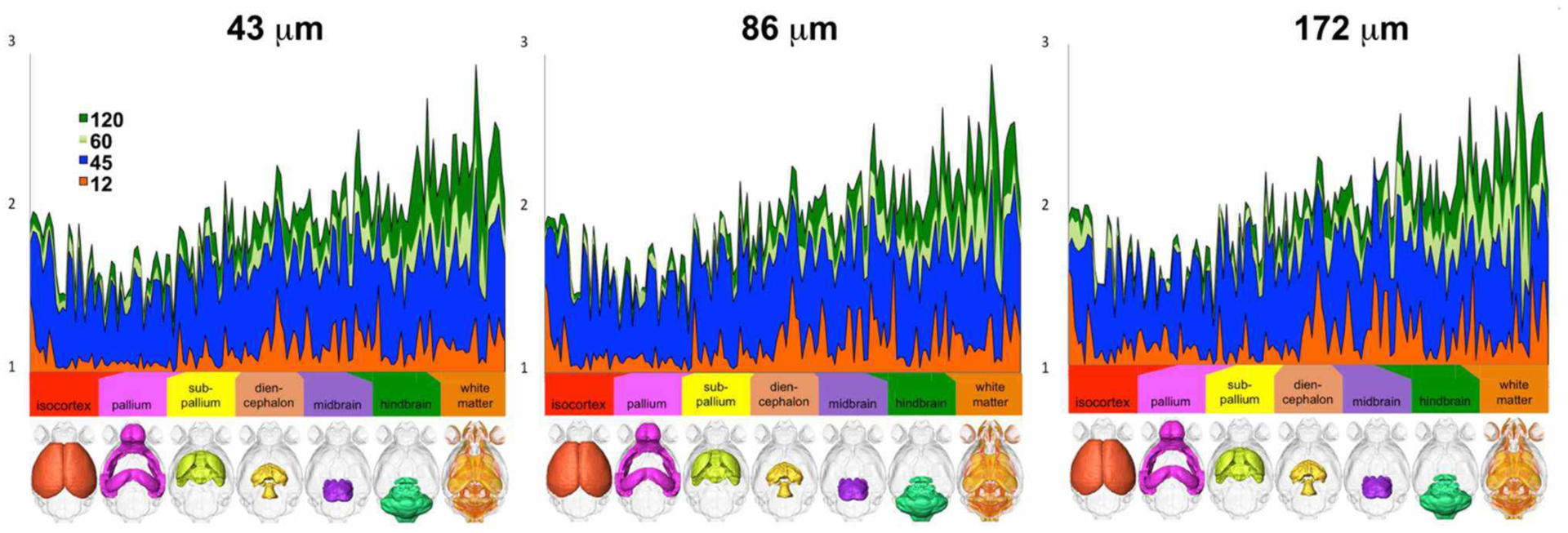
The number of voxels with one, two, and three dyads varied per region, but differences relative to the reference protocol (high-spatial, high-angular resolution) consistently decreased with increasing number of angular directions (only 12, 45, 60, and 120 directions are shown for simplicity) and with increasing spatial resolution (i.e., smaller voxels; ranging from 43-μm linear dimension for the reference to 86 and 172 μm).

These effects were found to be region dependent, although we noted a consistent trend in the ratios between one, two, and three dyads across all three resolution samples (Figure 3).

**FIGURE 4 |:**
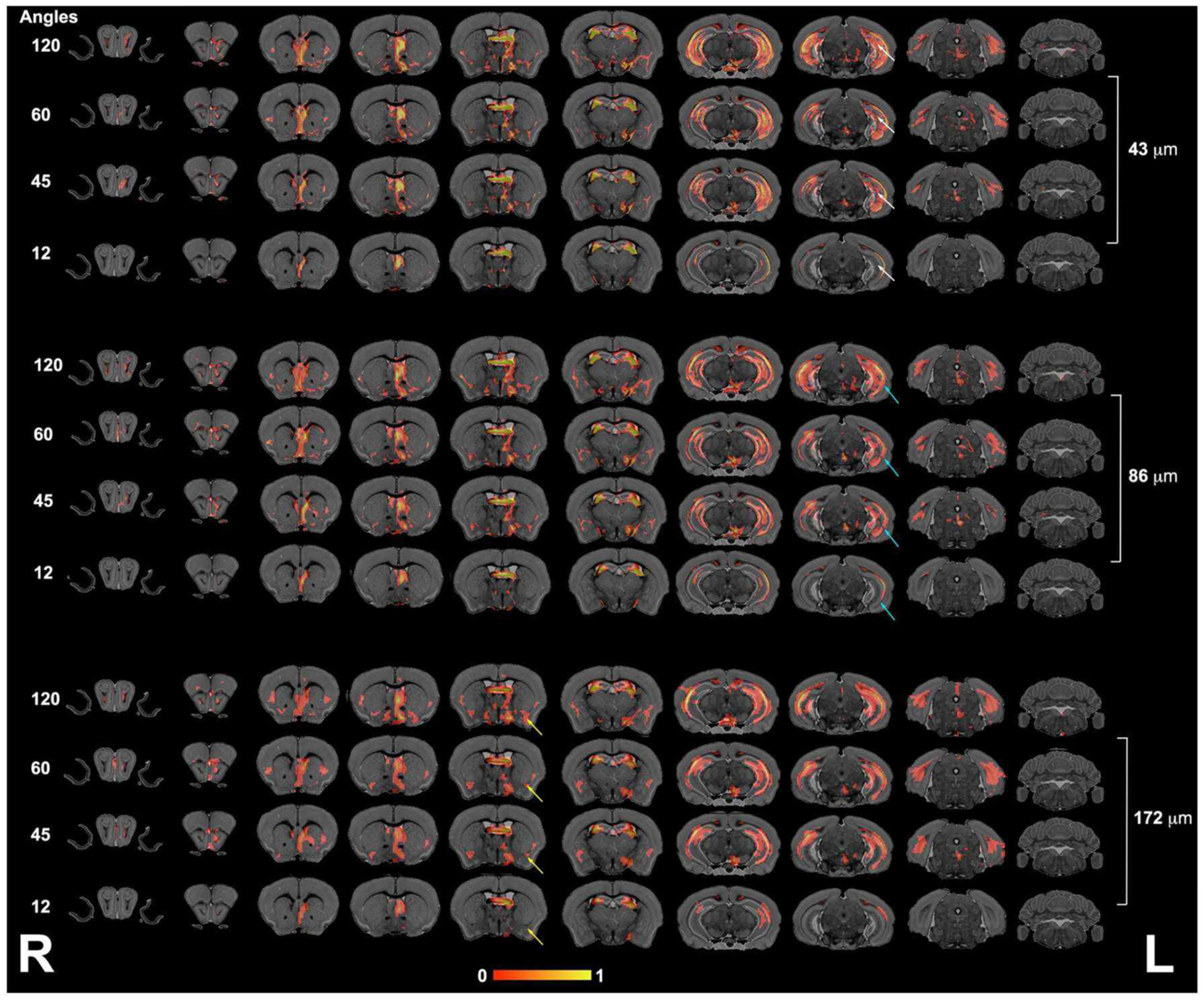
The fimbria connectivity mapped as a function of spatial and angular resolution. The connectivity of the left fimbria was reconstructed for protocols with 12, 45, 60, and 120 directions, at the full 43-μm resolution, clearly illustrating limitations of smaller angular sampling protocols at capturing projections though the hippocampus (white arrows) and amygdala (yellow arrows). Less clear were the effects of spatial resolution in the range 43–172 μm where SNR and partial volume effects both played a role. However, the projections through the hippocampus covered reduced areas, and projections into the alveus were partially lost in the lower resolution protocol (blue arrows). L, left; R, right.

We next focused our analysis on the fimbria and fornix because these tracts have been reported to be relevant in neurodegenerative conditions such as Alzheimer’s disease. Our qualitative evaluation showed that tract density maps reconstructed from 12 directions did not capture the cortical–cortical connectivity with the same sensitivity as the 45-, 60-, or 120-direction schemes (Figure 4). While less striking, the loss of spatial resolution also resulted in a change in the anatomical definition for the projections, and loss of connectivity through smaller regions (e.g., alveus).

**FIGURE 5 |:**
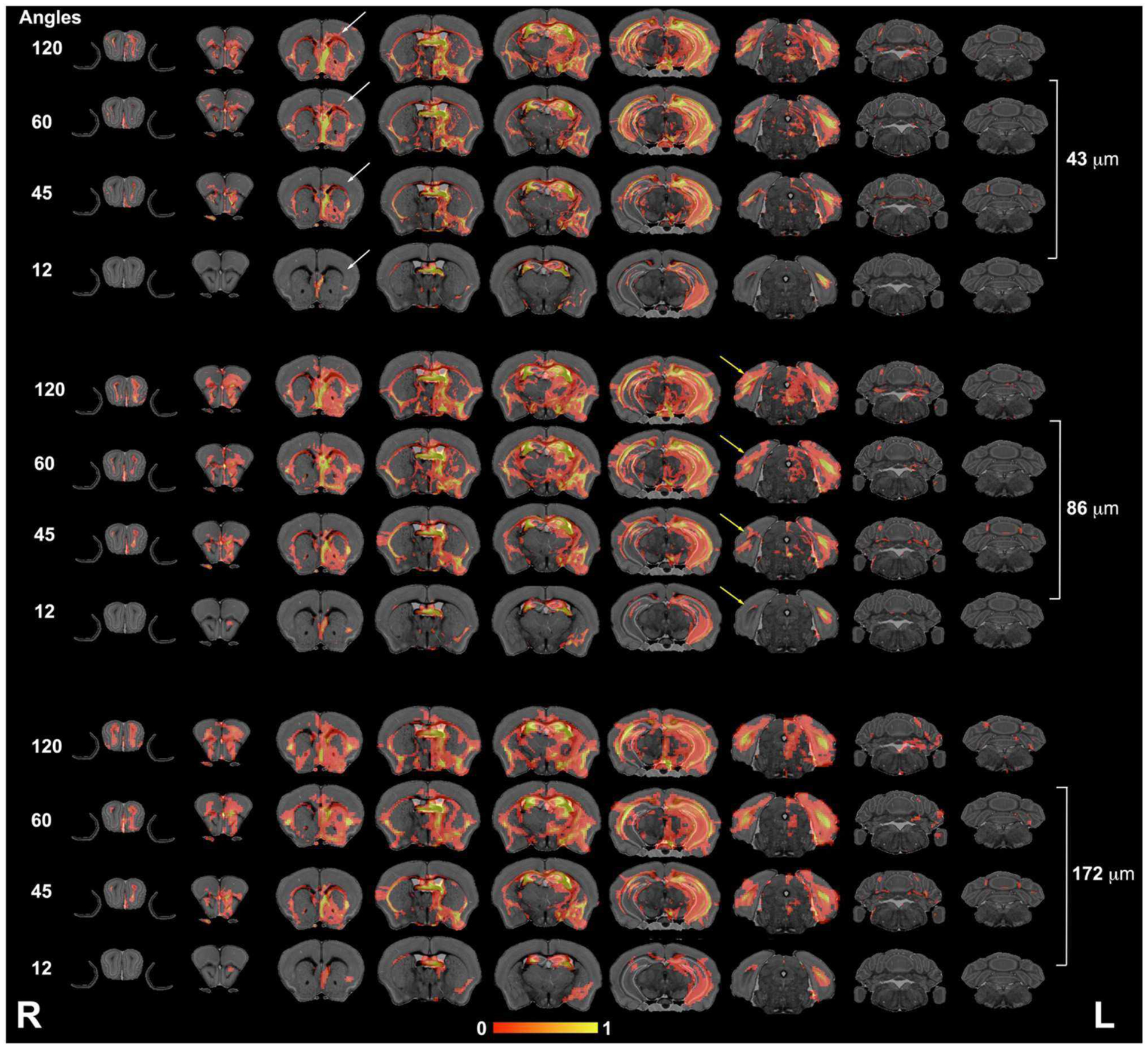
The hippocampus connectivity mapped as a function of spatial and angular resolution. Hippocampal connectivity (the left hemisphere was seeded) was affected more by angular resolution, especially for cortical regions (white arrows), but also in the ability to retrieve cross hemispheric projections (yellow arrows). L, left; R, right.

The connectivity of the left hippocampus (Figure 5) illustrated a striking similarity between the 120- and 60- directions schemes and loss of similarity for lower angular resolution schemes. In particular, cortical projections occupied smaller areas, especially those requiring interhemispheric connections. The loss of spatial resolution resulted in some clusters appearing larger (e.g., in the amygdala), or conversely, some of the connections were lost (e.g., hindbrain, cerebellum).

**FIGURE 6 |:**
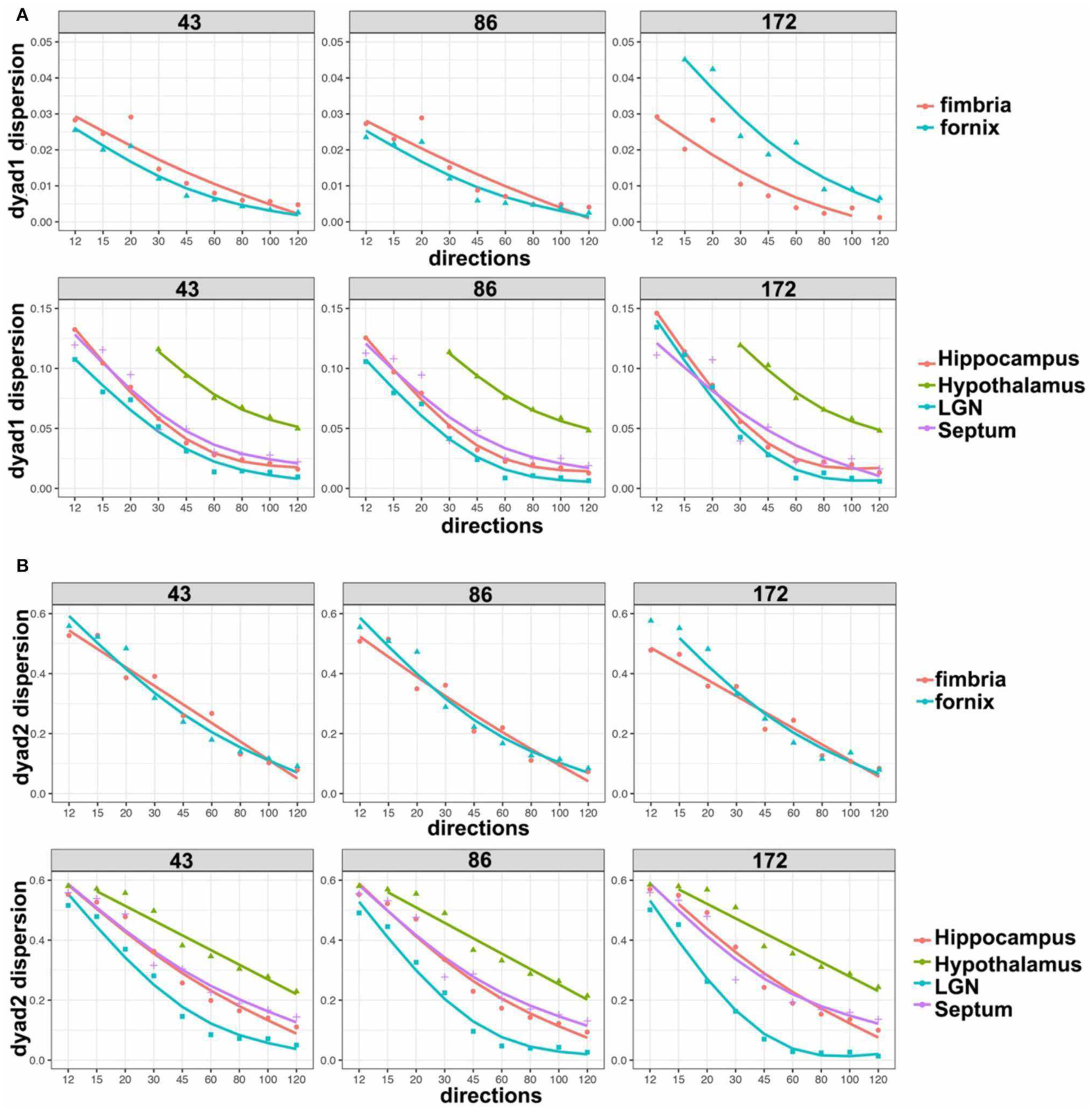
Dyad dispersion as a measure of error. The dyad one dispersion shows decreasing errors for higher angular samples, with an inflection point at 45^◦^ and increasing stability after 60^◦^ **(A)**. Similar errors were apparent for 43 and 86 μm, but the errors were larger for 172 μm. Dyad two showed a stronger effect of resolution, and larger errors, which also tapered off after more than 60 directions were acquired **(B)**. LGN, lateral geniculate nucleus.

We based our quantitative error analysis on the dyad dispersion for the first- (Figure 6A) and second-order dyads (Figure 6B). Our results showed that errors decayed as the number of angular samples increased both for white matter and for gray matter regions, and the errors increased as we relaxed the spatial resolution. This effect toward increased dispersion was particularly important for thin regions such as the fornix in dyad one (Figure 6A). An inflection point was noticed in particular for gray mater regions for 45 directions, and the curves tended to converge toward an asymptotic behavior starting with 60 directions. The dispersion values were larger for the second-order dyad (Figure 6B), but the trends were consistent with those observed for dyad one, with an asymptotic behavior predominantly observed in gray matter regions for schemes with more than 60 directions.

**FIGURE 7 |:**
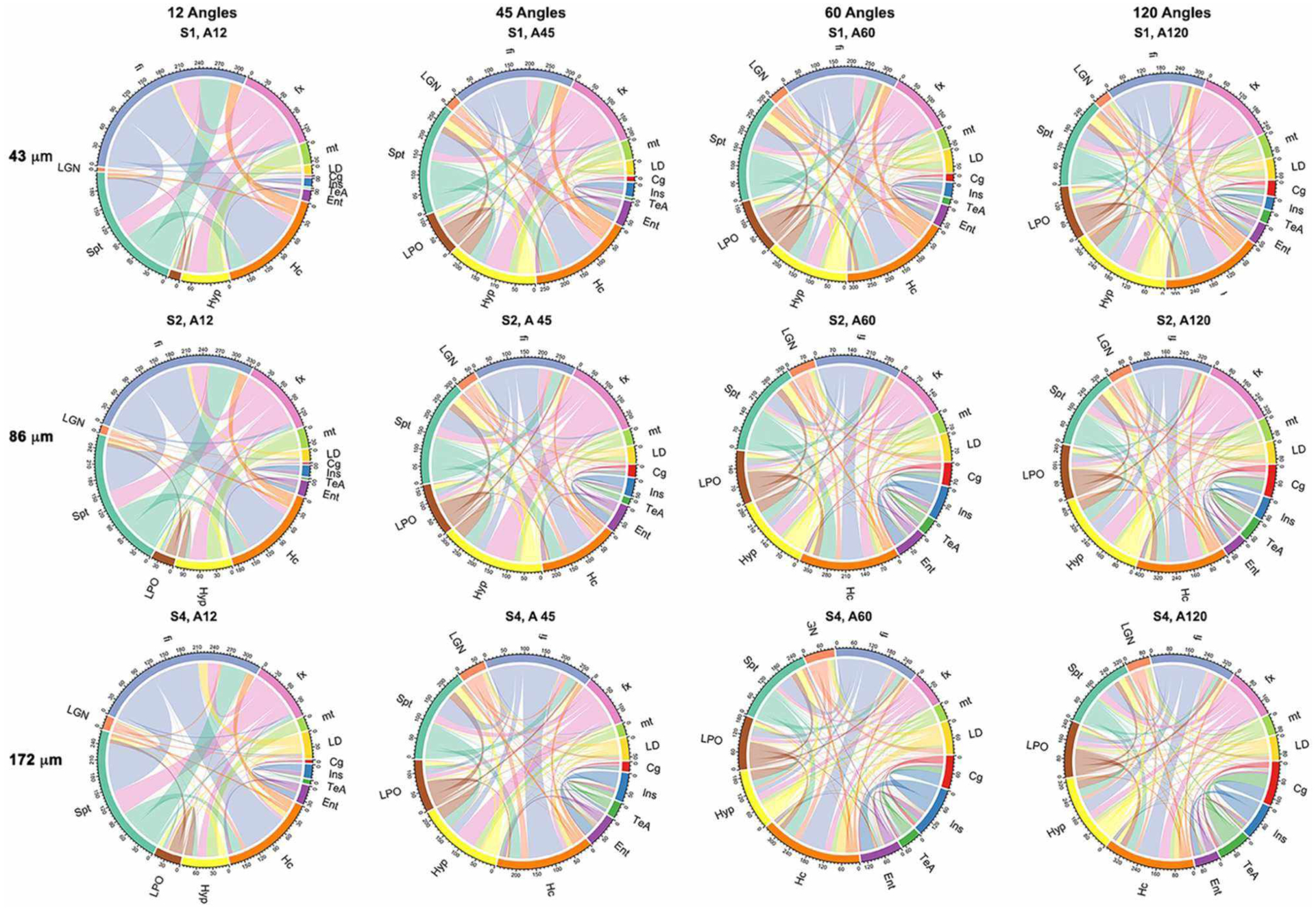
Adjacency matrices shown as chord diagrams for a subset of 13 regions relevant to neurodegenerative diseases showed qualitatively greater similarity to the reference connectome of 120 directions (A120) for 45 (A45) and 60 directions (A60) when compared to the similarity between 120 (A120) and 12 directions (A12). Spatial resolution also had an effect on the chord diagrams, in particular, at the level four times downsampling (S4; 172 μm), but this was less pronounced when comparing two times downsampling (S2; 86 μm) with fully sampled (S1; 43 μm) protocols. The chord diagrams showed that all protocols can capture the connectivity of the hippocampus (Hc) and its connecting fibers (fx, fornix; fi, fimbria), but shorter protocols have reduced sensitivity for smaller nuclei (LD, laterodorsal thalamic nuclei; LGN, lateral geniculate nuclei; LPO, lateral preoptic nucleus) and lack sensitivity required for cortical connectivity, e.g., for the cingulate (Cg) and entorhinal cortices (Ent).

Probabilistic tractography was used to generate connectivity matrices, based on the number of streamlines connecting selected regions that are likely to play a role in neurodegenerative disease models. In general, the connectivity differences between our reference and those of the 45- or 60-direction data sets were considerably smaller relative to those involving 12 directions (Figure 7). The sensitivity to capturing connectivity of smaller, and in particular, cortical gray matter regions was evident in the chord diagrams (Figure 7), which appeared sparser for 12 directions relative to 45, 60, and 120 directions. The sparsity was evident for gray matter-to-gray matter connections, in particular, for cortical domains. For small angular resolutions, the connectomes were dominated by wide bands involving myelinated white matter tract regions connecting to the hippocampus or septum. Gray matter-to-gray matter connections appeared more prominent with higher angular sampling, in particular for schemes with more than 45 directions, with a noticeable qualitative similarity between 60 and 120 angles.

To quantify the similarity between connectomes, we used non-parametric correlation analysis. The Spearman correlation among the connectivity matrices was significant (*p* < 0.001) for all comparisons tested.

**FIGURE 8 |:**
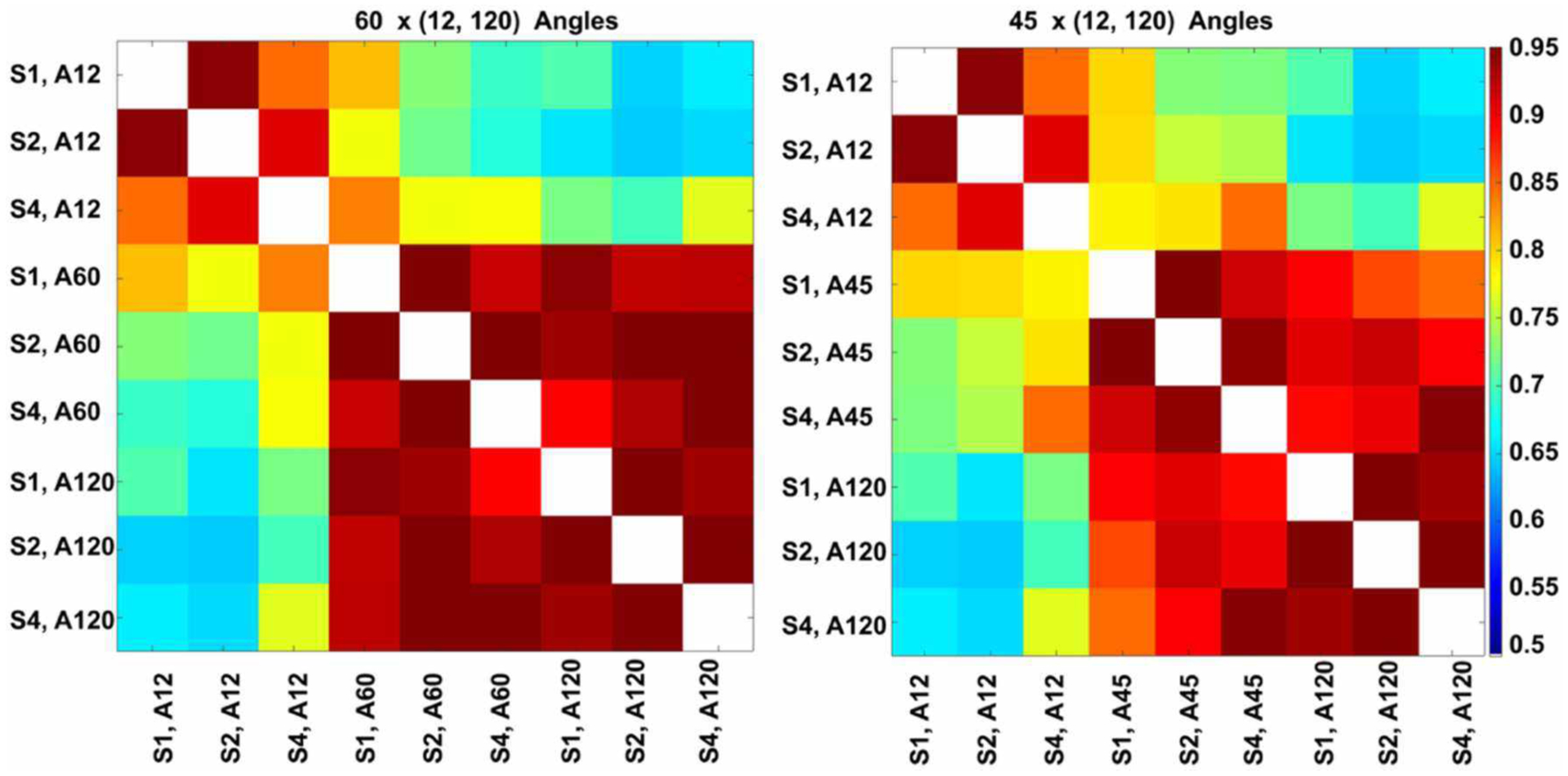
Global connectome similarity. Spearman correlations among the connectomes obtained for three resolution levels (S1, fully sampled at 43-μm linear voxel dimension; S2, downsampled by a factor of 2–86 μm; S4, downsampled by a factor of 4–172 μm; and each of the 12, 45, 60, and 120 angular resolution schemes (A12, A45, A60, A120) indicated that the highest similarities were obtained between the 60 and 120 angular samples schemes for all spatial resolutions. While the patterns were similar, similarities were lower for the 45 and 120 angular sample schemes for all resolutions relative to 60- and 120-direction schemes. When comparing against the reference set, the median was 0.89 for sets with 45 directions, but 0.92 for sets with 60 directions; and the maximum for 45 directions was 0.91, but 0.94 for 60 directions. The global maximum 0.97 was obtained when comparing (S2, A60) vs. (S2, A120); or (S1, A45) vs. (S2, A45).

The highest global correlation overall was found among the 86-μm spatial resolution connectomes acquired with 60 and 120 directions, 0.97, while the correlation between connectomes acquired at the same 86-μm spatial resolution with 45 and 120 directions was 0.92. When compared against the reference high-spatial, high-angular resolution protocol (S1, A120), the highest correlations were with (S2, A120) at 0.96, followed by (S1, A60) and (S2, A60) at 0.94, then (S4, A120) at 0.93 (Figure 8, left). When comparing the 45-direction protocols against the reference (high-spatial, high-angular resolution) protocol (S1, A120), the highest correlation was obtained with (S2, A45) at 0.91 followed by (S1, A45) at 0.90 (Figure 8, right).

In our experiments (S1, A120) was robust to downsampling, achieving a correlation of 0.96 with (S2, A120) and 0.93 with (S4, A120). The angular resolution had a strong effect, with the similarity dropping to 0.7 with (S1, A12), 0.9 with (S1, A45), and 0.94 with (S1, A60). The lowest similarity from our subgraph comparison was between (S2, A120) and (S2, A12) at 0.64, followed by (S2, A120) with (S1, A12) and (S2, A12) with (S4, A120) at 0.65. Our results suggest that higher-angular resolution schemes are generally more robust to detecting changes in animal models of neurological conditions.

**FIGURE 9 |:**
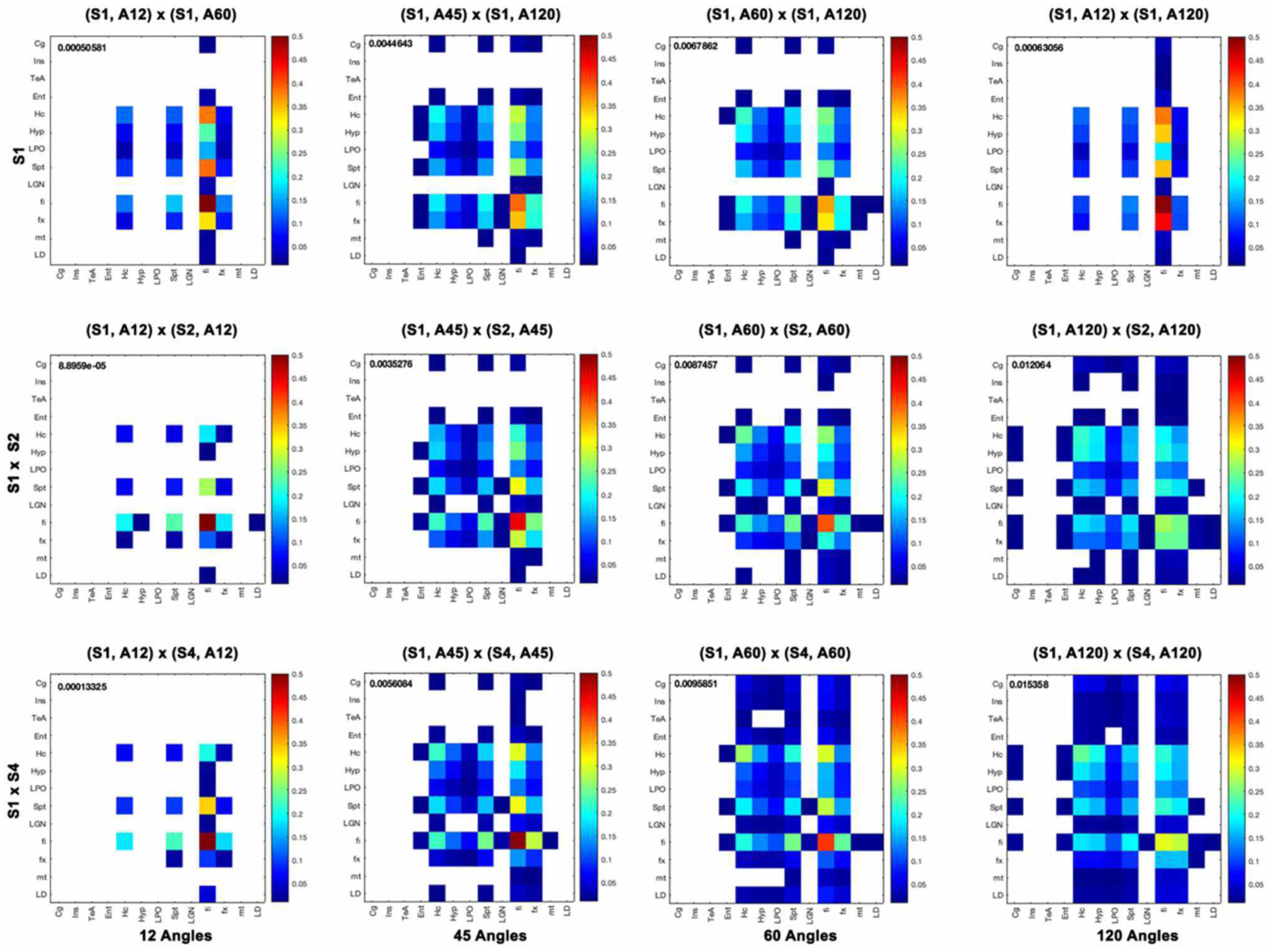
Vertex-based graph similarity showed that white matter tracts were more robust to downsampling relative to nodes representing gray matter and, in particular, cortical domains. Our subgraph’s regions of interest include the cingulate cortex (Cg), insular cortex (Ins), temporal association areas (TeA), entorhinal cortex (Ent), hippocampus (Hc), hypothalamus (Hyp), preoptic telencephalon (LPO), septum (Spt), lateral geniculate nucleus (LGN), fimbria (fi), fornix (fx), mamilothalamic tract (mt), and laterodorsal thalamic nuclei (LD). When comparing graphs for different spatial resolutions, the fi was most similar between 43- and 86-μm resolutions with a score of 0.85, and also relative to 172 μm with a score of 0.82, but most other graph nodes were not. Among angular sampling schemes, the fimbria was also robust, with a similarity score between the 12 and 120 directions of 0.5, followed by the fornix, hippocampus, and septum with 0.1. The cortical regions were most likely to be dissimilar when changing spatial or angular sampling schemes. Median similarity scores are shown for each graph comparison.

Furthermore, we computed measures of similarity between individual graph vertices. The vertex-wise graph similarity pairwise for these subgraphs, shown in Figure 9 after thresholding at a 0.1 level, illustrated differences in sparsity. Graphs were less sparse as the numbers of angular samples/directions increased above 45 to 60 and 120 directions. Based on the vertex score, the robustness of some nodes relative to others became evident when undergoing downsampling, first for fimbria, followed by the fornix. The similarity between the hippocampus (Hc) and fimbria (fi) and fornix (fx) was 0.1 and 0.07. For the 45-direction scheme, the similarity between full resolution and 86-μm resolution for fimbria was 0.45, while for the fx, it was 0.18, and for the hippocampus and septum, it was 0.16 indicating more robustness for larger regions. Hc with fi and fx was 0.21 and 0.11, respectively. The similarity for fi (0.27) and fx (0.24) decreased with downsampling spatial resolution for the 120-direction schemes; however, more regions picked up more similar connections across these two graphs. For example, the hippocampus score was 0.21, and for septum, 0.15, with Hc to fi and fx being 0.17 and 0.12, respectively. These results suggest that while major white matter tracts were robust with respect to changes in spatial and angular resolution, this needs to be balanced against the vulnerability of smaller white matter tracts and cortical domains to such changes, and sparsity of the connectome and graph similarity matrices are important factors to consider.

In conclusion, our results support that angular and spatial resolution need to be balanced with respect to time- and cost-imposed demands to enable population studies, and parameters for efficient protocols with minimum loss of sensitivity should be recommended, suggesting that an acquisition with 60 angular samples provides a good compromise in terms of minimizing errors relative to a reference high-spatial, high-angular resolution protocol. Halving both spatial and angular resolutions yields an eight-time speed factor. When time is a constraint, compromises in terms of spatial resolution may need to be made. Importantly, more efficient acquisitions are amenable to future population studies.

## DISCUSSION

Several studies using tractography-based connectomics have reported abnormal network organization associated with morphometric changes and AD pathologies [26, 27]. Still, to understand the etiology of human neurodegenerative diseases such as AD has proven difficult. This is due to AD’s complex nature, multiple pathologies, and various associated comorbidities. In spite of their limitations and simplicity, mouse models provide tools to examine the effect of singular pathologies, and the interaction of multiple factors in a well-controlled environment and, importantly, from early on.

Given the challenges in phenotyping mouse models of neurological conditions, imaging protocols are required to provide sensitive quantitative biomarkers with good spatial mapping abilities. In a previous study, we have defined a diffusion MRI data reference set with the highest reported resolution for the whole mouse brain [11]. This data set was acquired using 120 angular samples and 43-μm spatial resolution, requiring a scan time of 235 h. Replicating this acquisition for population studies is prohibitive in terms of both time and cost. To address this problem, we must define reduced protocols that ensure sufficient fidelity and compare well with the reference data set, but that can be acquired in a greatly reduced time. This will enable population studies and give insight into vulnerable networks that may provide early biomarkers and enable us to quantify disease progression or response to interventions. We, thus, performed a simulation study, examining subsets of angular samples from the reference set, and the effect of reducing spatial resolution.

We have focused on evaluating regions of interest in Alzheimer’s research, from hippocampus, which represents 5% of the mouse brain volume, to fimbria and fornix, which represents only 0.05% of the mouse brain volume [13]. In our simulations, we have extracted nine gradient subsets to evaluate subsequent changes in several metrics. Our metrics evaluated the stability/noise of the diffusion schemes through the condition number; the relative number of detected voxels with one, two, three, and four directions or dyads, globally and on a per-region basis; the extent of projections for the fimbria and hippocampus; the dyad dispersion and connectome similarity globally, and on a per-node basis. Our results corroborated to support that schemes with 45 angular samples started to approximate well the reference connectome and that errors were substantially reduced and started plateauing for 60 angular samples acquired with 43- or 86-μm spatial resolution.

The performance of the 60-direction protocols was very similar to the reference set, both qualitatively and quantitatively, and executing such protocols would lead to substantial reductions in acquisition time (eight times when combined with a downsampled spatial resolution), providing a connectome with 0.94 correlation with the reference set. Further reductions in acquisition times come from compressed sensing [28], which several groups have implemented for mouse MRI [29–31]. Such advances can help translate diffusion protocols into population studies [32] incorporating multiple biomarkers from morphometry [33], microstructural properties based on diffusion [13] or magnetic susceptibility [34], or network properties [35]. Such integrative studies may better predict changes in behaviors, modeling those observed in humans with neurodegenerative conditions [34].

Optimization studies are important when it comes to assess newly proposed diffusion models, acquisition schemes, various pathologies, and different animal models—to help understand the human brain [36, 37]. In this study, we were limited to a single specimen meta-analysis using extracted gradient subsets, but this approach provided useful information to help devise future group-based analyses. We have used the condition number for our acquisition schemes as a measure of noise performance [38], but better schemes may be designed in the future to increase rotational stability. Our recommendations are based on a finite set of metrics for a subgraph selected based on relevance to AD and a single b value acquisition. More regions would need to be included to generalize the recommendations; however, our results point to robustness of major white matter tracts, in contrast to thin, small white matter tracts and cortical–cortical connections, which are particularly vulnerable to reduced acquisition schemes. Moreover, future studies may be improved by the addition of multiple diffusion shells and more complex diffusion models. A reduction by a factor of eight in acquisition time for half the resolution and 60 directions provided an attractive avenue. However, the number of voxels with more than one dyad could not be retrieved to the same extent at lower spatial resolution. Still the similarity of the subgraphs based on anatomical regions known to be involved in pathological aging approached 0.94. Our study benefitted from the use of high-performance computing, which enhances the speed of optimization and quality control studies required before establishing new protocols in both mice and humans [39].

Our methods are applicable to other DWI studies in rodent models, and we expect that the conclusions will remain valid in the same range of spatial resolutions and may change as we approach resolutions used in human brain imaging. Still, our results align with recommendations for human brain studies, where angular sampling schemes with more than 30 directions [40] are recommended for a robust estimation of the diffusion tensor orientations and mean diffusivity. More than 45 directions are required for spherical deconvolution using spherical harmonics of order eight [41, 42]. Acquiring more than 45 directions helps with fitting and avoiding issues with imperfections in the uniformity of the diffusion gradient directions and meet signal-to-noise requirements. Therefore, 40 directions or more are widely used for the human brain [26, 27], and state of the art protocols employ multiple diffusion shells/b values, e.g., *n* = 3, and with 60 directions per shell [43]. Harmonization efforts [44] are currently under way to establish diffusion imaging guidelines and/or translate among protocols, such as those used for ADNI3 [45].

While the field of mouse imaging is much smaller, and efforts for standardization are not yet widespread, we hope that our study can inform the design of future experiments using statistical connectomics in models of neurological conditions, such as Alzheimer’s disease, and that network biomarkers will provide enhanced sensitivity to early and subtle changes arising due to multiple, interacting pathologies.

## Figures and Tables

**TABLE 1 |:** The smallest mean condition numbers (CN) ranked the 120-direction scheme as optimal, followed by the 60, then 45-direction schemes.

Angles	CN	CN (mean)	STD	STD (CN)/SQRT (Angles)	Rank CN (mean)	Rank STD
12	1.647	1.661	3.626E–02	1.047E–02	8	7
15	1.709	1.659	4.779E–02	1.234E–02	7	9
20	1.696	1.675	4.758E–02	1.064E–02	9	8
30	1.633	1.639	1.799E–02	3.285E–03	6	3
45	1.610	1.615	2.180E–02	3.250E–03	3	4
60	1.561	1.604	2.685E–02	3.467E–03	2	5
80	1.645	1.621	2.877E–02	3.217E–03	5	6
100	1.643	1.618	1.761E–02	1.761E–03	4	2
120	1.585	1.584	7.494E–04	6.841E–05	1	1
